# Internal Disorders of Mango Fruit and Their Management—Physiology, Biochemistry, and Role of Mineral Nutrients

**DOI:** 10.3390/plants13182596

**Published:** 2024-09-17

**Authors:** Muhammad Asad Ullah, Amit Khanal, Priya Joyce, Neil White, Andrew Macnish, Daryl Joyce

**Affiliations:** 1 School of Agriculture and Food Sustainability, The University of Queensland, Gatton Campus, Brisbane, QLD 4343, Australia; a.khanal@uq.net.au (A.K.); daryl.joyce@daf.qld.gov.au (D.J.); 2 Independent Researcher, Karalee, QLD 4306, Australia; 3 Department of Agriculture and Fisheries, Leslie Research Facility, Toowoomba, QLD 4350, Australia; 4 Department of Agriculture and Fisheries, Maroochy Research Facility, Nambour, QLD 4560, Australia; andrew.macnish@daf.qld.gov.au; 5 Department of Agriculture and Fisheries, Gatton Research Facility, Gatton, QLD 4343, Australia

**Keywords:** calcium, disorders, internal breakdown, quality, *Mangifera indica* L., mango, nitrogen

## Abstract

Mango (*Mangifera indica* L.) is a popular fruit grown in tropical and subtropical regions. Mango has a distinctive aroma, flavour, and nutritional properties. Annual global mango production is >50 million tonnes. Major producers of mango include India, Bangladesh, China, Mexico, Pakistan, Indonesia, Brazil, Thailand, and the Philippines, and it is shipped worldwide. Harvested mango fruit are highly perishable, with a short shelf life. Physiological disorders are among the major factors limiting their postharvest quality and shelf life, including when fruit need phytosanitary treatments, such as hot water treatment, vapour heat treatment, and irradiation. This review focuses on problematic physiological disorders of mango flesh, including physiology and biochemistry. It considers factors contributing to the development and/or exacerbation of internal disorders. Improved production practices, including pruning, nutrient application, and irrigation, along with monitoring and managing environmental conditions (viz., temperature, humidity, and vapour pressure deficit), can potentially maintain fruit robustness to better tolerate otherwise stressful postharvest operations. As demand for mangoes on international markets is compromised by internal quality, robust fruit is crucial to maintaining existing and gaining new domestic and export consumer markets. Considering mango quality, a dynamic system, a more holistic approach encompassing pre-, at-, and post-harvest conditions as a continuum is needed to determine fruit predisposition and subsequent management of internal disorders.

## 1. Introduction

Fruit quality is a suite of valued characteristics that augment worth. Perceived quality differs from the perspectives of producers and consumers. As key characteristics of fruit quality, producers typically favour enhanced yield, disease resistance, and postharvest life, including low susceptibility to disorders. On the other hand, consumers are interested in fruit flavour and visual appeal [1]. Reduced shelf life, skin damage, lenticel spotting, off-flavours, postharvest diseases (e.g., anthracnose, stem-end rot) [2], and flesh breakdown are problems that compromise fruit quality [3]. They negatively affect fruit mouthfeel, visual appeal, marketability, and overall consumer satisfaction.

Physiological disorders affecting commercially important mango cultivars include blacktip [4], fruit splitting [5], internal necrosis [6], and internal breakdown [7]. Internal breakdown disorders encompass specific symptoms that include soft nose [8], spongy tissue [9], stem-end cavity [10], jelly seed [11], and internal browning [12]. Internal disorders (IDs) often lack external manifestation, which renders them problematic to detect [13]. IDs were first reported in Indian mango export consignments destined for Europe in 1932–1933 by Cheema and Dani [14]. Such disorders are still prevalent and pose a significant challenge for the mango industry across the globe [15,16,17,18,19,20].

Diverse predisposing factors have been associated with IDs, including management/cultural practices, environmental and edaphic factors, fruit characteristics (e.g., size, weight, density/specific gravity), and nutritional imbalance [11,21,22]. Nutrient deficiency is considered a key factor contributing to pre- and/or postharvest physiological dysfunction [9]. Measures to identify susceptible fruit are sought for a better understanding of the causes of physiological disorders and their prevention.

This literature review covers key physiological and biochemical changes associated with major mango internal disorders. It also overviews factors that contribute to their expression and identifies research gaps in the interactive factors towards better-informed management of IDs.

## 2. Common Internal Disorders of Mango

Major IDs of mango fruit include soft nose, jelly seed, stem-end cavity, flesh cavity, flesh browning, and spongy tissue.

**Figure 1:**
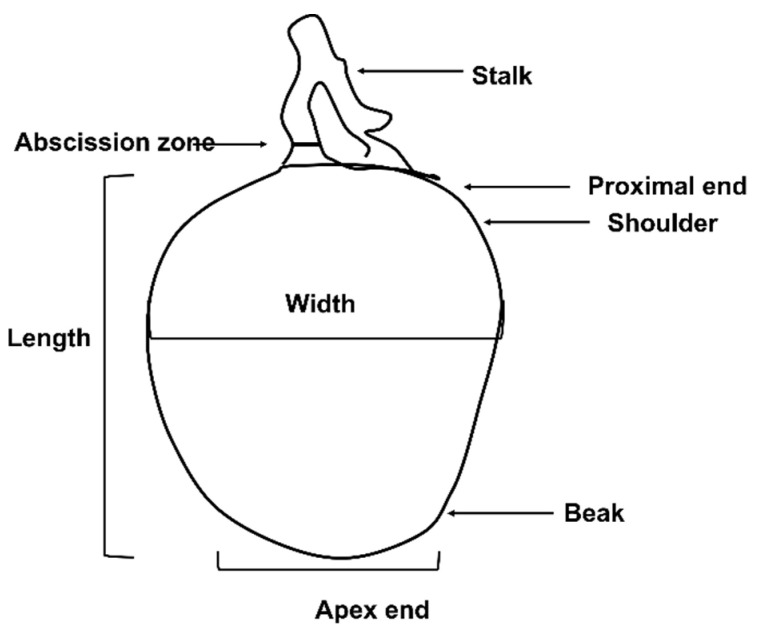
Characteristic morphological features of the mango.

**Figure 2:**
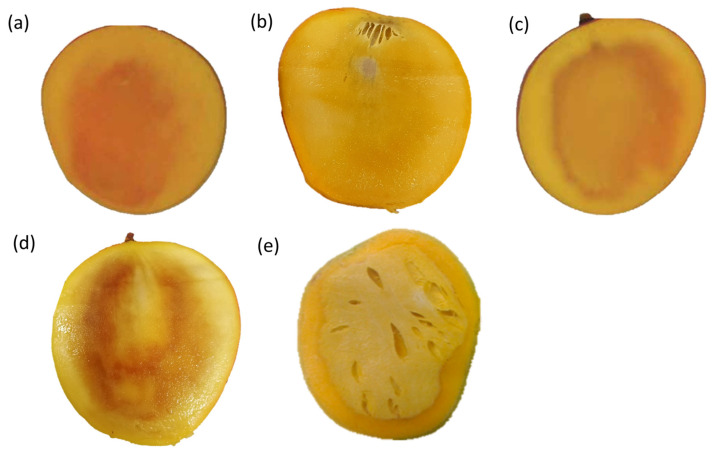
Internal disorders in cv. ‘B74’ mango: (**a**) Soft nose [40], (**b**) stem-end cavity [41], (**c**) jelly seed [40], (**d**) internal flesh browning [41], and (**e**) spongy tissue in cv. ‘Alphonso’ [9].

- Soft nose is characterized by flesh softening and yellowing at the fruit apex due to mesocarp breakdown [23] (Figure 1 and Figure 2a). The ‘beak end’ of the fruit appears over-ripe, indicating rapid localised ripening [24]. Soft nose may be assessed by touching the beak end, it being softer than the rest of the fruit [25]. Soft nose incidence is both cultivar-dependent and less prevalent in alkaline soils than in acidic sandy soils [26].
- Stem-end cavity is characterized by cavity formation in the proximal area between the peduncle and the mesocarp as vascular tissue deterioration (Figure 1 and Figure 2b) [10]. In the early stages of development, tissues at the proximal end of affected fruit turn brown, followed by cavity formation and necrosis [25]. In advanced stages, necrosis around the cavity manifests in the internal mesocarp. Symptoms are like spongy tissue and jelly seed. Microscopic analysis reveals xylem deterioration, procambium damage, and calcium oxalate crystal accumulation in the cavity [10].
- Jelly seed appears as flesh breakdown in the mesocarp around the fruit seed/endocarp (Figure 2c). It manifests as a jelly-like mass around the fruit stone. Complete tissue disintegration leads to tissue browning and the appearance of watery translucent areas in extreme cases [27]. Jelly seed onset symptoms vary among cultivars. ‘Van Dyke’ and ‘Tommy Atkins’ showed jelly seed after 8 weeks of fruit set [10]. By contrast, symptoms in ‘Irwin’ appeared 12 weeks after fruit set [10]. Harvesting fruit early is recommended to mitigate jelly seed incidence [28].
- Internal flesh browning is an important physiological disorder of fleshy fruit (Figure 2d). It is typically associated with long-term storage-related physiological changes in fruit [29,30,31]. Flesh browning is considered a result of cell wall and membrane degradation. Concomitant release and mixing of enzymes are associated with browning (viz., polyphenol oxidase and phenylalanine ammonia-lyase) and phenolic substrate compounds. The enzymes and phenolics are usually localised in different cellular compartments to prevent their mixing. Upon cell wall weakening and degradation, phenolic compounds oxidise to coloured O-quinones [32]. Cell wall weakening is often associated with low Ca concentration in fruit mesocarp [33]. Lo’ay and Ameer [34] discerned positive effects of ascorbic acid-blended Ca nanoparticles for the alleviation of internal flesh browning in low-temperature stored ‘Hindi Be-Sennara’ mango by inhibiting cell wall degrading enzymes, such as pectinase and cellulase.
- Spongy tissue disorder is common in Indian mango cultivars, particularly ‘Alphonso’. Affected tissue displays symptoms like soft nose and jelly seed, with spongy mesocarp, off-odour, and pale-yellow or off-white flesh, with or without cavities (Figure 2e) [35,36]. Early studies implicated nutritional imbalance (e.g., high nitrogen and low calcium) and environmental factors (e.g., rain and field temperatures) in this disorder [36,37]. Dysfunction ultimately resulted in a shift from aerobic to anaerobic respiration, including an acid pH shift, cell wall degradation, and synthesis of free radicals [38].

Similarities among symptoms of jelly seed, spongy tissue, stem-end cavity, and soft nose disorders suggest some commonalities. Raymond et al. [10] concluded that all commence with cellular disorganisation and cell wall degradation. Nevertheless, temporospatial distribution of symptoms in different parts of the fruit distinguishes each disorder; hence, their specific symptomology.

The progressive expression of internal visual symptoms makes their early detection difficult [39].

## 3. Anatomy and Biochemistry of Internal Disorders

Macroscopic differences have been observed among IDs based on the spatial distribution of symptoms and their visual appearance. Information in the literature on the anatomy of IDs in mango is otherwise limited. Generally, however, cell disintegration and cell wall rupture appear to be the first indication of disorder onset. However, histological studies suggest differences among cultivars affected by different disorders. Torres and Saúco [8] observed severe xylem deterioration and cell wall disintegration in the internal breakdown-affected mesocarp of highly susceptible ‘Tommy Atkins’ as compared to less susceptible ‘Lippens’, which showed translucent mesocarp with intact cell walls.

Raymond et al. [10] characterised early symptoms of stem-end cavity in cultivars ‘Tommy Atkins’, ‘Irwin’, and ‘Van Dyke’ as tannin deposition in resin ducts and xylem that leads to the discolouration of flesh around the cavity. The continuous accumulation of tannins eventually causes toxicity and necrosis in the advanced stages of stem-end cavity disorder development [10]. Ca accumulates in the stem-end cavity in the form of oxalate crystals and creates a localised deficiency in the cells because it is not available for biochemical functions even though total pulp Ca concentrations may be high [10,42]. This vascular disruption markedly limits the nutrient supply to inner mesocarp cells and weakens the flesh cellular integrity proximal to the seed.

Biochemical changes associated with physiological disorders in mango fruit have been studied by Chitarra et al. [33] and De Oliveira Lima et al. [43]. Mesocarp of spongy tissue-affected ‘Alphonso’ mango fruit showed increased pectin methyl esterase and malic enzyme activities [44] and reduced invertase and amylase activities. A study on ‘Dashehari’ and ‘Langra’ mangoes revealed higher activities of polymethyl esterase and cellulase in jelly seed-affected flesh [45]. A recent transcriptomic study revealed abscisic acid interacted with ethylene to accelerate starch decomposition into sugars and promoted soft nose incidence in cv. ‘Keitt’ mango [46].

Enzymes associated with biochemical changes in mango flesh affected by various IDs are presented in Table 1.

Previous studies also indicated the role of gas exchange in the development of IDs [47]. In particular, the shift in metabolic processes from aerobic to anaerobic is due to the low availability of oxygen inside mango fruit [48]. Consequently, cell membrane integrity may diminish and result in internal quality defects, including cavities, flesh gelation, and tissue browning. Over-mature mango fruit tend to accumulate more anaerobic metabolites, such as acetaldehyde and ethanol, in fruit flesh over time [49]. The accumulation of such metabolites in muskmelon fruit has been associated with reduced sugar content and water-soaked tissue appearance [50]. These metabolites can be used as biochemical markers to predict the predisposition of fruit to disorders, such as for internal browning in pears under storage conditions [51,52]. 

A similar response in late-harvested litchi fruit compared to early-harvested under modified atmosphere storage has been reported [53]. This was ascribed to fermentation leading to flesh deterioration. Reduced mitochondrial activity due to membrane damage may render cells incapable of producing sufficient energy to sustain normal cell activity [54]. 

**Table 1:** Enzyme activities in mango flesh afflicted by various internal disorders.

Cultivar	Disorder	Enzyme	Activity	Reference
‘Alphonso’	Spongy tissue	Superoxide dismutase, catalase, peroxidase, polyphenol oxidase	Lower	[55]
‘Tommy Atkins’	Spongy tissue	Amylase	Lower	[56]
‘Alphonso’	Spongy tissue	α-amylase, invertase	Lower	[35]
‘Tommy Atkins’	Spongy tissue	Peroxidase, polyphenol oxidase	Higher	[43]
‘Alphonso’	Spongy tissue	Pectin methyl esterase, malic enzyme	Higher	[44]
‘Chiin Hwang’	Jelly seed, lumpy tissue, soft nose,	α-amylase activity	Lower	[57]
‘Langra’	Jelly seed	α-amylase, pectin methyl esterase, cellulase, polyphenol oxidase	Higher	[45]
‘Dashehari’	Jelly seed	α-amylase, pectin methyl esterase, Cellulase, polyphenol oxidase	Higher	[45]
‘Amrapali’	Jelly seed	Pectin methyl esterase, pectate lyase, polygalacturonase	Higher	[58]

## 4. Factors Affecting Fruit Quality and Robustness

Fruit quality attributes are largely determined and/or influenced by preharvest factors and early fruit development conditions [59]. Fruit susceptibility to physiological disorders depends upon cultivar type, untoward environmental variability, poor management practices, and postharvest handling conditions [60]. Table 2 presents the range of factors that directly and/or indirectly influence fruit quality and physiology.

### 4.1. Role of Mineral Nutrients in Fruit Flesh

An imbalance of mineral nutrients, such as nitrogen (N), calcium (Ca), potassium (K), magnesium (Mg), and boron (B), is influential in fruit susceptibility to IDs (Table 3). Many studies conclude that low Ca, high N, and a high N/Ca ratio in fruit flesh predispose fruit to IDs [57,60,61,62,63]. There are also conflicting reports implicating fruit flesh K, Mg, P, and B in disorders (Table 3).

Considering Table 3, the following discussion focuses on Ca and N in the internal quality of mango fruit. It explores their transport, accumulation, and analysis in the soil–plant–fruit continuum. Also, considering variability across cultivars and regions, there are no generic threshold values for element ratios as indices for fruit quality [8,64,65].

**Table 3:** Mineral nutrients associated with increased (↑) or decreased (↓) expression of some major internal disorders in mango flesh. ‘↑/↓’ denotes associations with both increased and decreased defect expression.

Defect	N	Ca	K	P	Mg	B	N/Ca	Reference
Spongy tissue	↑	↓			↑		↑	[35,61,66,67]
Jelly seed	↑	↓					↑	[57,61,62]
Internal flesh breakdown	↑	↓	↑/↓	↑	↑/↓	↓	↑	[7,8,18,44,64,68,69]
Soft nose	↑	↓			↓		↑	[24,57,70]

The balance between nutrients putatively potentiates fruit robustness at harvest and its subsequent inherent ability to withstand the rigours of postharvest handling [63]. Innate fruit robustness generally cannot be improved after harvest. It is, therefore, important to focus on the best management strategies for ensuing preharvest periods. Studies of preharvest N and Ca application to internal fruit breakdown are collated in Table 4. Applications of N and Ca nutrient solutions reduced internal breakdown in the ‘Tommy Atkins’ mango, such that the incidence was relatively low when both flesh N and Ca were high versus high N and low Ca [8]. Silva et al. [71] applied foliar Ca as CaCl_2_ during fruit development in ‘Tommy Atkins’. Their analysis of peel and flesh tissues at eating ripe revealed an inverse correlation between Ca and internal breakdown incidence. Although not necessarily or always representing causation, correlations of fruit flesh N and Ca have been associated with the incidence and severity of IDs. Such studies often do not necessarily take important aspects of elemental distribution and sampling variability of N and Ca analysis into consideration, which are hereby discussed.

**Table 4:** Mineral nutrition and internal breakdown in mango cultivars.

Cultivar	Disorder	Nutrient	Source	Method	Time of Application	Results	Reference
‘Sensation’	Spongy tissue, Jelly seed	N, Ca	NH_4_NO_3_, CaSO_4_·2H_2_O	Drip irrigation	Continuous throughout growing season	↑N = ↑Disorder ↑Ca = ↓Disorder	[61]
‘Van Dyke’	Jelly seed	Ca	CaCl_2_, Ca(NO_3_)_2_	Foliar	At fruit set	↑Ca = ↓Disorder	[62]
‘Tommy Atkins’	Internal flesh breakdown	N, Ca	-	Drip irrigation	Continuous throughout growing season	↑N = ↑Disorder ↑Ca = ↓Disorder	[68]
‘Sensation’	Internal flesh breakdown, Stem-end cavity	Ca, Mg, K, B	CaCl_2,_ MgCl_2_, KCl, Solubor^®^ (20% B)	Foliar	Weekly from 3 weeks after fruit set to 3 weeks before harvest	No effect	[69]
‘Sensation’	Internal flesh breakdown, Stem-end cavity,	Ca	Ca-EDTA	Stem injection	One month before harvest	↑Ca = ↓Disorder	[69]
‘Chaunsa’	Soft nose	Ca, Mg	CaCl_2,_ CaSO_4_, Ca(OH)_2_, MgCl_2_	Foliar	2 weeks before harvest	↑Ca = ↓Disorder ↑Mg = ↓Disorder	[72]
‘Keitt’	Watery pulp breakdown	N, Ca	NH_4_NO_2_, CaSO_4_·H_2_O	Soil	Pre- and post-flowering	No effect	[64]

“↑” calcium, nitrogen, and magnesium in the results column indicate a higher concentration in flesh in response to their application. “↑ or ↓ disorder” indicates an increase or decrease in disorder expression, respectively.

#### 4.1.1. Calcium

Ca is critical in fruit development, cell wall strengthening, and signal transduction pathways [73]. Its deficiency is associated with increased fruit susceptibility to physiological disorders [70,74,75]. These include bitter pit in apples [76], blossom end rot in tomatoes [77], and cork spot and end spot in pears and avocadoes, respectively [78,79,80]. Ca deficiency in mango fruit can weaken mesocarp cell walls resulting in jelly seed [10] and spongy tissue [81] disorders. Ca import and accumulation in fruit varies spatiotemporally during development [82,83,84]. Given the importance of Ca in fruit development and signal transduction, its availability, concentration, and transport to specific cells need to be regulated physiologically and managed in a cultivation context [85,86]. Excess Ca can result in cell wall rigidity and cellular phytotoxicity [42,87]. Ca deficiency due to low availability and/or limited transport can result in cell membrane breakdown (e.g., leakiness) and disorders (e.g., flesh browning) [88].

##### Calcium Translocation

**Figure 3:**
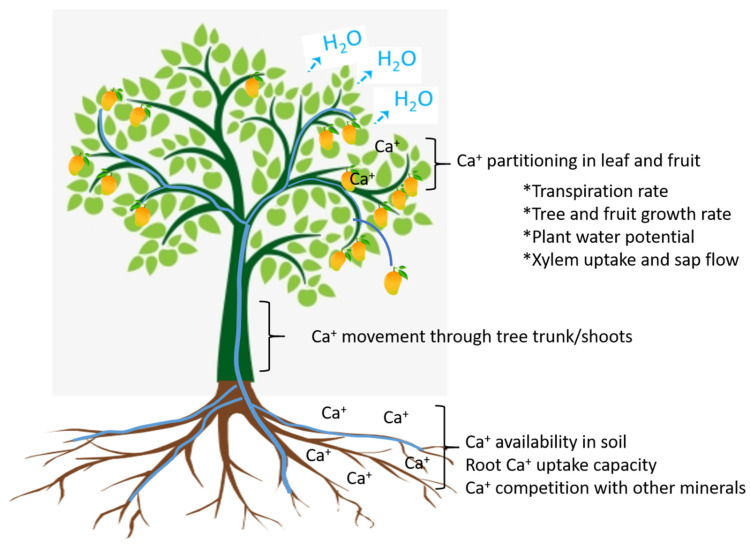
Processes and their influences on calcium uptake, translocation, and partitioning in fruit trees. ‘*’ indicate key factors involved in Ca calcium partitioning at branch level.

The uptake, translocation, and accumulation of Ca within the plant canopy among its leaves and fruit is mediated by the factors depicted in Figure 3. The final amount of Ca in fruit depends on the complex interplay of availability in the soil, uptake through roots, including competition with other minerals in the root system, mobility in xylem sap, and competition between vegetative (i.e., foliage) and reproductive (viz., fruit) sinks (Figure 3). Ca in the soil can be tightly bound to negatively charged particles or soluble in the soil solution. Plant roots can only take up Ca present in the soil solution [89,90]. The quantity of Ca in the soil solution is influenced by edaphic factors and can change with soil pH and type of fertiliser application. Root uptake is modulated by its growth rate and transfer into xylem vessels via symplastic and apoplastic pathways [91,92]. Ca availability in interaction with other cations (e.g., K, Mg) affects uptake rate and movement along the soil–root–plant continuum [90]. These cations tend to compete with Ca for uptake through roots. Thus, a balance in terms of optimal cation exchange capacity is desirable to facilitate Ca uptake during critical fruit growth periods [63].

Ca loaded into xylem vessels is transported towards the shoot by the mass flow of water due to more negative water potential in the canopy as generated by growth and transpiration [93]. Ca partitioning within the canopy and between leaves and fruit is modulated by xylem cation exchange capacity, xylem sap Ca content, leaf and fruit growth rates and transpiration into the surrounding air [42]. Leaves are relatively strong sinks that tend to accumulate more Ca due, by virtue of their high surface area to volume ratio and stomatal frequency, to greater transpiration than fruit [94]. Fruit generally exhibit a relatively high transpiration rate during early development stages which then diminishes as they approach harvest maturity [82,83]. As fruit reach maturity, their relative water uptake also decreases due to loss of xylem functionality, stomatal differentiation into lenticels, and wax deposition on fruit cuticular surfaces [95,96,97].

Ca also acts as a secondary messenger facilitating abscisic acid, auxin, and gibberellic acid signalling, which regulate biochemical, cellular, and morphological functions, including fruit set and cell division and expansion up to and during fruit ripening and senescence [42,98]. Ca can also influence phytohormones that modulate its distribution at the tissue level. Auxins are pivotal in developmental pathways, including fruit set, cell division, and expansion processes, which involve Ca as a secondary messenger [99]. Auxin export from developing tissues increases Ca partitioning [100,101,102]. Exogenous application of the auxin transport inhibitor (2,3,5 tri-iodobenzoic acid; TIBA) reduced the uptake of Ca during fruit development in tomato [103] and avocado fruit [104]. High light-induced hydroxycinnamic acid levels in kiwifruit also increased Ca uptake by reducing auxin degradation [105].

Tonetto de Freitas et al. [106] reported that exogenous application of abscisic acid enhanced Ca accumulation by increasing xylem sap flow to fruit, resulting in lesser susceptibility to blossom-end rot in tomatoes. Abscisic acid may redirect Ca flow towards fruit by reducing stomatal conductance in leaves. Foliar application of abscisic acid increased Ca partitioning into and lessened bitter pit in apple fruit [107]. Transcriptional analysis of the response to foliar abscisic acid application revealed the down-regulation of MdCAX, MdACA8, and MdCDPK, three genes involved in the regulation and partitioning of Ca in apples [107].

##### Monitoring of Calcium in Mango Fruit

Sampling

To ascertain fruit mineral status, it is advised to collect fruit from the middle part of the tree to secure a representative sample and to avoid higher and lower levels in the canopy [76,108,109,110]. Ferguson and Triggs [108] suggested analysing fewer fruit from many trees rather than many fruit from fewer trees with a view to obviate fruit-to-fruit variability. The sampling strategy is an important factor to consider for minimising variability and realising a truly representative estimation of nutrient levels. In apple fruit, for example, Ca levels in individual apple fruit from one tree may vary two- to three-fold [111].

Fruit mesocarp analysis has typically been used to indicate fruit Ca status. However, differences within whole fruit are highly evident amongst local tissue Ca levels [112]. Temperature differences across fruit may exceed ca. 10 °C between sun-exposed and shaded sides of avocado fruit [113]. Hence, sun-exposed tissue may have a different physiology (e.g., transpiration rate) as compared to other regions of the fruit. Ca is mobile within the xylem, and its partitioning within the fruit depends to a degree on the local transpiration rate. Similar temperature differences of ~5 °C between sun-exposed and shaded mango fruit were reported by Katrodia and Sheth [114] and again may differentially influence local tissue Ca levels.

A typical means of sampling fruit flesh for mineral analysis is to use fruit corer to take samples from skin to seed along the equatorial region [64]. However, the skin tends to accumulate more Ca than the inner flesh. Joyce et al. [82] found 0.371 mg/g DW Ca in the skin versus 0.095 mg/g DW Ca in the inner mesocarp of the ‘Kensington Pride’ mango. Burdon et al. [70] analysed Ca in mangoes ‘Kent’ and ‘Beverly’ and found that the outer mesocarp had a higher Ca content (9.52 and 7.21 mg/100 g FW, respectively) as compared to the inner mesocarp (5.00 and 4.51 mg/g FW, respectively). Similarly, longitudinal spatial variation in Ca accumulation is seen from the proximal to distal ends of the fruit. The proximal top of the ‘Kensington Pride’ mango had higher Ca (0.088 mg/g DW) levels as compared to the distal end (0.052 mg/g DW) [82]. Burdon et al. [70] also reported such variation for Ca from stem-end to apex in ‘Sensation’ fruit. In ‘Alphonso’, Gunjate et al. [115] reported variations in Ca from 122 to 110 mg/100 g DW between stem-end and apex, respectively. Localised Ca deficiency in the inner mesocarp and the distal part of the fruit may cause internal breakdown and soft nose, respectively [24,70,116]. Overall, a general trend is observed in the spatial variation of Ca accumulation of skin > outer mesocarp ≥ middle mesocarp > inner mesocarp (Table 5) [82,117]. This variation could be due to physiological differences in cell type, size, and Ca movement from the inner mesocarp to the outer mesocarp through vascular bundles, at least partially as directed by transportational mass flow.

It is difficult to compare Ca concentrations among different reports due to the limited information provided, for example, regarding regions of the fruit sampled and/or units of measurement, including that converting mg/g FW to mg/g DW is problematic without known moisture content of the tissue sampled. In this context, it is advisable to specifically and consistently state the sampling position for these analyses in predicting postharvest quality and/or the expression of physiological disorders.

**Table 5:** Calcium concentrations reported in different parts of various mango cultivars.

Cultivars	Skin	Outer Flesh	Middle Flesh	Inner Flesh	Proximal Flesh	Distal Flesh	Reference
‘Beverly’	-	7.21 mg/100 g FW	-	4.51 mg/100 g FW	-	-	[70]
‘Dashehari’	4570 mg/kg dw	-	-	460 mg/kg dw	-	-	[118]
‘Glen’	1900 mg/kg dw	-	-	700 mg/kg dw	-	-	[119]
‘Haden’	2300 mg/kg dw	-	-	700 mg/kg dw	-	-	[119]
’Irwin’	3600 mg/kg dw	-	-	600 mg/kg dw	-	-	[119]
‘Keitt’	3900 mg/kg dw	-	-	1000 mg/kg dw	-	-	[64]
‘Kent’	-	9.52 mg/100 g FW	-	5.00 mg/100 g FW	-	-	[70]
‘Kent’	3600 mg/kg dw	-	-	900 mg/kg dw	-	-	[119]
‘Kensington Pride’	523 mg/kg dw	123 mg/kg dw	55 mg/kg dw	50 mg/kg dw	94 mg/kg dw	71 mg/kg dw	[82]
‘Kensington Pride’	1670 mg/kg dw	-	-	580 mg/kg dw	-	-	[120]
‘Kensington Pride’	1740 mg/kg dw	560 mg/kg dw	400 mg/kg dw	410 mg/kg dw	-	-	[117]
‘Kensington Pride’	2400 mg/kg dw	-	-	600 mg/kg dw	-	-	[119]

##### Calcium Fractions in Fruit

Ca in fruit tissues can be subdivided into various fractions based on solubility, availability, and biochemical or physiological activity. Exchangeable Ca (e.g., adsorbed on proteins and pectin) and soluble Ca (e.g., associated mainly with nitrates, chlorides, and organic acid) are generally considered active forms as compared to tightly bound forms, like Ca oxalate, carbonate, and phosphates [42,121,122]. Ca deposited in the form of oxalate crystals in the parenchyma of developing fruit showed low or no solubility and mobility [42]. The presence of insoluble Ca may create localised deficiencies of physiologically active Ca, which can lead to physiological disorders [10].

Pavicic et al. [123] determined that bitter pit development in apples was associated with low soluble Ca concentrations in affected fruit. Studies on cracking resistant and susceptible cultivars of litchi determined higher levels of Ca present in the cell walls of the pericarp of resistant cultivars [124,125]. However, the presence of Ca-rich crystalline bodies in cracking susceptible litchi cultivars, as detected by X-ray microanalysis, suggested that Ca deficiency might be due to the presence of the insoluble Ca form rather than available Ca [126]. Therefore, in reporting associations with physiological disorders, it is desirable to state the Ca form or pool being analysed.

#### 4.1.2. Nitrogen 

##### Sources and Translocation

N is an essential component of amino acids, proteins, enzymes, and chlorophyll. It plays a key role in cell division during the early growth of young tissues, including leaves, buds, and flowers, particularly at the onset of flowering and fruit set [127,128,129,130,131]. N is highly mobile and present mostly as proteins in cells [130]. Fruit trees acquire N for vegetative growth and reproduction by uptake through the roots and by internal N cycling. In trees experiencing N deficiency, proteins in older cells undergo proteolysis, and the resultant amino acids move to newer cells [132]. Proteolysis results in a decline in leaf chlorophyll content and attendant chloroplast collapse. Hence, N deficiency is typically evident as yellowing in older leaves.

Roots take up N in both nitrate (NO_3_^−^) and ammonium (NH_4_^+^) forms. However, NO_3-_ is the preferred source in well-aerated soils [133]. Both forms are usually derived from applied mineral fertilisers and/or native N mineralization. After absorption, N is translocated to different plant organs. Trees may not respond to N fertilisers due to built-up reserves in perennial organs from previous years [127,128,131,134]. Documentation of seasonal changes in the composition of amino acids in the xylem sap in apple, grape, and cherry allows N remobilisation and recently absorbed N to be distinguished [135,136]. N^15^-labelled fertilisers have been used to follow the fate of recently applied and stored N in fruit trees. N reserves from the previous year are generally used to support tree N demand and vegetative growth in the following year. Remobilisation depends on the amount of N stored, tree size, and age. In apples and other deciduous trees, typically there is intense N withdrawal from ‘autumn’ leaves for translocation to perennial root, trunk, and stem storage organs [127,131,137]. According to Niederholzer et al. [138], about 50% of the N is translocated out of the leaves of peach trees before their abscission. This N is stored in the tree trunk or roots. Upon either remobilisation from storage or root uptake, N is preferentially allocated to newly developing vegetative and reproductive organs [139].

##### Effect of Nitrogen on Fruit Quality

An increase in N supply promotes tree vigour and, if managed carefully, can enhance yield [129,140,141]. Limited availability slows tree growth and adversely affects crop load [130]. On the other hand, excess N supply can negatively affect postharvest fruit quality [142]. The optimum N levels mediate fruit skin colour, size, yield, and flavour [60].

Fruit N concentration is generally high during early development stages and decreases thereafter. Fruit response to N application is influenced by timing, method, rate, source, tree phenological stage, and edaphic and climate conditions [143]. Increased tree vigour in response to high N supply typically leads to higher leaf-to-fruit ratio and fruit size, and lower fruit Ca concentrations [144,145]. Positive correlations between fruit size and occurrence of IDs have been reported for mango [146,147] and other fruit, such as avocado [148,149].

Young [23] reported that increased N fertilisation was associated with increased soft nose incidence in the ‘Kent’ mango. Inconsistencies in later studies were ascribed to unaccounted-for seasonal effects [147]. Murthy [44] also questioned the pivotal involvement of Ca and/or N in the development of internal disorders with findings on the ‘Alphonso’ mango, suggesting that high P and low K were responsible for pulp tissue breakdown. However, Tarmizi et al. [65] discerned higher N/Ca in the ‘Haramanis’ mango with a greater incidence of insidious fruit rot. In mango fruit ontology studies from fruit set to ripening, Raymond et al. [21] found no difference in mineral composition between healthy fruit and fruit with disorders. However, Torres et al. [68] determined a positive correlation between internal breakdown and N concentration in the ‘Tommy Atkins’ fruit mesocarp and an inverse relationship with fruit Ca concentrations. Similarly, Ma et al. [7] found higher N and lower Ca concentrations when comparing flesh mineral concentrations for healthy and ‘Keitt’ mangoes showing disorders.

How high the N concentration must be to contribute to IDs in mango fruit remains unclear. One possible explanation is that high vegetative growth in response to excess N redirects Ca flow to leaves, rendering developing fruit vulnerable to Ca deficit; the latter is potentially exacerbated by a concomitant increase in fruit size leading to Ca dilution in the flesh [149,150]. It follows that rapidly growing fruit with limited Ca supply are more prone to Ca-mediated physiological disorders during postharvest handling and storage [79,80,144,151].

Such studies and findings suggest that optimising N is as important as Ca supply to achieve robust, high-quality, long shelf-life fruit [68]. Improved understanding of the complex interplay between orchard management practices and preharvest mineral analyses regarding postharvest fruit quality, storage, and shelf life potential is enabling the industry to make more informed decisions.

In an experiment to determine the long-term effects of N application on apple fruit quality, Fallahi et al. [141] determined that the optimum leaf N content should be in the range of 2.05–2.30% for acceptable quality in ‘Fuji’ apples. Similarly, studies on mango orchards in Spain stipulated that trees with leaf N 15–18 g/kg DW and Ca 17–20 g/kg DW were prone to fruit internal flesh breakdown and deterioration [146].

Mineral analyses of leaves have been extensively used in the industry to predict outturn fruit quality [152,153]. However, other studies question their reliability due to poor predictive potential and suggest that fruit mineral analysis is the more reliable diagnostic tool for quality prediction [154,155,156,157]. If fruit can be characterised by industry as likely to be high or low in certain quality attributes, then it should help with making more informed decisions to enhance profitability [158]. In this context, the appropriate timing of leaf and possibly inflorescence and/or immature fruit sampling is an important consideration [159].

### 4.2. Irrigation and Fruit Quality

The timing and amount of rainfall and irrigation play a prominent role in fruit growth and sizing [160,161]. Stress due to water deficit from flowering up to halfway through the mango fruit rapid cell division period markedly affects growth rate and final fruit size [162]. Due to lower cell number, an overall 34% reduction in fruit size was reported by Simmons et al. [163] for water-stressed ‘Kensington Pride’ mango trees as compared to non-stressed fruit. Thus, with a lower cell number, limited water supply may lower Ca concentrations in developing fruit and can adversely affect postharvest quality attributes.

On the other hand, an oversupply of water in the lead up to fruit maturity during and after the cell expansion phase leads to increased fruit size, yield, and also susceptibility to physiological disorders [160,164]. Late development phase fruit expansion leads to Ca dilution, which negatively affects postharvest fruit quality in terms of susceptibility to IDs [144,165].

Deficit irrigation studies on pears, apples, and mangoes returned mixed effects on postharvest fruit quality during critical growth phases [166,167,168,169]. Nonetheless, managing fruit size through best management practice irrigation could prospectively help manage fruit Ca and overall quality.

### 4.3. Temperature

Temperature modulates fruit metabolism, growth, maturation, and ripening [170,171,172]. Both preharvest and postharvest storage temperatures contribute to the final fruit quality experience of consumers, including the expression of physiological disorders [173].

Preharvest temperatures during fruit development cannot easily be controlled. However, they can be monitored such that measures can be taken to maintain fruit robustness and quality [174]. Orchard temperatures predominantly affect fruit physiology by influencing water loss through transpiration in association with prevailing vapour pressure deficits [175,176]. Transpiration-mediated water loss influences fruit growth rate, diurnal shrinkage, and final quality at harvest [177,178], including fruit size, taste, and aroma [178,179]. Orchard temperatures can potentially be managed by installing shade netting and/or overhead sprinkler cooling systems to mitigate the adverse effects of extreme temperatures [180,181,182].

The internal temperature of the growing fruit reflects the energy exchange associated with fruit metabolic activity, energy conductance, and evapotranspiration. Collectively, these define the heat budget of a fruit [183]. Tertiary factors affecting a fruit’s heat budget include environmental factors, like temperature, humidity, airspeed, solar radiation, and physical properties, such as fruit volume, skin colour and reflectance, pulp density, and skin permeability across developmental stages [184].

To maintain quality, optimum postharvest handling, treatment and storage temperatures and durations at each step beyond harvest are also equally important. Exposure to elevated postharvest temperatures, especially for relatively prolonged durations, can hasten ripening, inactivate enzymes, inhibit protein synthesis, and alter membrane integrity. In adversely affected fruit, an increase in reactive oxygen species predisposes fruit quality deterioration, including flesh discolouration [185], internal breakdown, and cavity formation [186,187]. Moreover, tropical mango fruit are sensitive to low-temperature storage. Maintaining a balance between storage time and temperature for specific harvest lots helps preserve quality and longevity [188].

### 4.4. Fruit Maturity and Postharvest Implications 

Optimum fruit maturity at harvest is important for postharvest quality, shelf life, and consumer preferences [189]. Fruit maturity is defined as physiological maturity and commercial maturity. A fruit that has attained maximum growth and development stage is physiologically mature. Commercial maturity is the stage of fruit development when consumers’ preferred characteristics have been attained in a commodity [190]. ‘Desert’ mango varieties must be harvested green mature to ripen fully. ‘Salad’ cultivars are harvested and consumed ‘green’ for culinary purposes. Physiologically mature mangoes are harvested before the onset of their ethylene and/or respiratory climacteric rises [191].

Early harvest of physiologically immature desert fruit develop poor flavour and are more susceptible to mechanical damage and physiological disorders (such as flesh browning and flesh cavities) during postharvest operations, like vapour heat treatment (VHT), hot water treatment (HWT), and irradiation [49,192,193]. On the other hand, fruit harvested over-mature can exhibit off-odours, poor texture, and short shelf life. The severity of IDs, such as jelly seed and watery pulp breakdown in mango [64] and flesh browning in avocado [194], is characteristically high in over-mature fruit.

## 5. Control Measures and Recommendations to Minimise Internal Disorders

Mineral nutrient levels change throughout fruit development stages. Young and Miner [195] recommend maintaining <1.2% leaf N and >2.5% leaf Ca concentrations prior to flowering with a view to minimising susceptibility to disorders. Hofman and Whiley [191] developed a best management practice guide for the ‘B74’ mango and recommended maintaining N at 1.0–1.5% and Ca at 2.0–3.5% in young mature leaves at the bud swelling stage for optimum quality fruit. Maintaining a balance between N and Ca levels is considered important to achieve optimum fruit quality. Antagonistic effects of N fertilisation on Ca uptake and accumulation in fruit have been researched in various fruit crops [61,196,197]. Depending on pH, lime or gypsum are recommended to increase Ca levels in low and high-pH soils, respectively, to improve the cation exchange percentage in the soil [198].

Subraman et al. [199] found IDs in mango fruit to be more prevalent in the coastal areas of India compared to inland orchards, possibly due to prevailing high humidities. Gunjate et al. [200] suggested that prolonged sun exposure following harvest could alter fruit physiology in favour of the expression of disorders. Katrodia and Rane [201] presented a heat convection theory that fruit from lower branches were more likely to develop physiological disorders due to heat convection from soil to surrounding air near the lower branches of the canopy, which results in high-temperature conditions. In this regard, orchard management practices such as mulching can lower soil temperatures and reduce the incidence of IDs [114,202].

**Figure 4:**
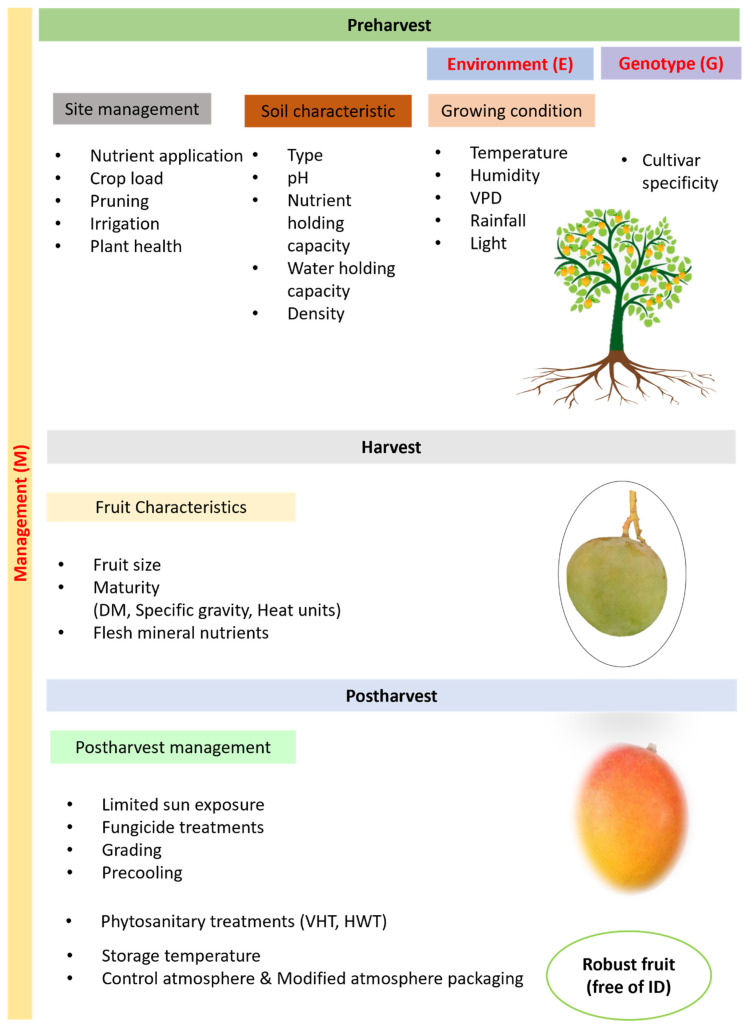
Major considerations at preharvest, harvest, and postharvest stages across the ‘genotype × environment × management’ spectrum to produce robust mango fruit.

In a complex interplay, various other factors can influence fruit quality. For example, fruit weight and density are potential contributing factors (Figure 4). ‘Alphonso’ mango fruit harvested at 1.0–1.2 relative density were less likely to manifest IDs than fruit at >1.2 relative density [22]. Harvesting at the green mature stage is recommended to reduce IDs, but it may result in poorer eating quality in some cultivars. As late-harvested fruit are more susceptible to internal flesh browning, an immediate shift to cooling chambers after harvest is recommended to preserve quality and shelf life [192,193,203]. Noticeable differences have been observed in the susceptibility to and tolerance of IDs in different cultivars of mango [41,61,204]. Selecting compatible rootstock and scions could be another way to reduce disorders, as certain rootstocks can also improve nutrient uptake and accumulation in fruit [205,206].

## 6. Conclusions

Fruit robustness at harvest and postharvest quality and shelf life are predominantly linked to preharvest nutrient status.

- Nutrient imbalances, like high fruit N and low Ca, can negatively affect fruit quality and the incidence of postharvest disorders. Increasing Ca concentrations in fruit flesh is a challenge, as applying more Ca to the soil does not guarantee its accumulation in fruit.
- Controlling tree vigour by pruning, managing crop load by thinning, carefully timing irrigation, and small frequent nutrient applications can potentially improve Ca levels in fruit, especially during the early developmental stages when the Ca accumulation rate is high.
- While N manipulation is achievable due to its mobility, application strategies must limit fruit N without negatively affecting fruit development and yield.
- Careful monitoring of soil, leaf, and fruit nutrient status is recommended and desirable.
- When sampling and analysing fruit and leaf samples, adopting recommended strategies to minimise variation and avoid ambiguity in results is advised. Other factors, including temperature, humidity, light radiation, and vapour pressure deficit, are likely to differentially influence fruit development and physiology.
- Considering complex interplays of variables, the consistent production of robust, quality mango fruit requires a holistic approach. Mango fruit quality is an output of a dynamic system that integrates influences of the growing environment, genotype, and associated management practices at preharvest, harvest, and postharvest stages.

## 7. Future Prospects

Towards producing more robust mango fruit (free of internal disorders), a ‘systems thinking’ approach could be implemented for fruit quality that uses quality as the system’s output to map how multiple factors pre-harvest and post-harvest interact to influence robustness status. Studies in the literature most often consider absolute mineral concentrations as a function of fruit robustness. However, minerals are present in different physiological forms. For instance, Ca is available in plant cells in different fractions, viz., soluble Ca (physiologically active form), cell wall-bound, and oxalate crystals. Future research should consider this aspect as it could inform which type of Ca fertiliser may prove to be more useful in raising soluble Ca levels in the mesocarp cells during critical fruit growth and development stages. Future research should also explore rapid, efficient, and cost-effective benchtop/on-farm analysis tools. These can markedly improve crop quality through the timely and efficient monitoring of fertiliser application. It also can help generate extensive datasets to better understand the dynamic roles of these nutrients in horticultural crops.

## Figures and Tables

**Table 2:** Preharvest factors affecting mango fruit quality.

Genotype	Cultivar (Rootstock, Scion)
Environment	Soil type (pH, cation exchange capacity, water holding capacity) Temperature (heat injury, chilling injury) Moisture (rain, relative humidity, precipitation) Radiation (day-length, wavelength, intensity)
Management	Canopy management (pruning, thinning, plant growth regulators) Nutrition (calcium, nitrogen, potassium, boron) Irrigation (time, frequency) Harvest (maturity, timing, method)

## Data Availability

Not applicable.
